# The effect of esketamine on emergence delirium in pediatric patients undergoing general anesthesia: a meta-analysis of randomized controlled trials

**DOI:** 10.3389/fphar.2025.1616843

**Published:** 2025-06-09

**Authors:** Shiyue Li, Yue Li, Pengfei Gao

**Affiliations:** State Key Laboratory of Oral Diseases, National Clinical Research Center for Oral Diseases, Department of Anesthesiology, West China Hospital of Stomatology, Sichuan University, Chengdu, China

**Keywords:** esketamine, emergence delirium, general anesthesia, pediatric, meta-analysis

## Abstract

**Background:**

The aim of this study was to investigate the effect of esketamine on emergence delirium in pediatric patients.

**Methods:**

We searched Pubmed, Cochrane Controlled Register of Trials, and Embase from inception to December 2024. Studies were independently evaluated for inclusion criteria and exclusion criteria by two reviewers. The primary outcome was the incidence of emergence delirium during the post-anesthesia period. The secondary outcomes were the PAED scores, FLACC scores, PACU stay time, and the incidence of nausea and vomiting.

**Results:**

Ten studies including 853 children were eligible for this meta-analysis. The pooled data revealed that esketamine administration significantly reduced the incidence of emergence delirium in pediatric patients (RR: 0.40, 95% CI: 0.30–0.53, P < 0.00001, I^2^ = 4%). Compared with the control group, esketamine also displayed lower PAED scores (MD: -3.66, 95% CI: -5.85–1.47, *P* = 0.001, I^2^ = 99%) and FLACC scores (MD: -2.47, 95% CI: -3.32–1.61, *P* < 0.0001, I^2^ = 89%). Esketamine had no significant effect on the PACU stay time (MD: 0.5 min, 95% CI: -1.51–2.51, *P* = 0.63, I^2^ = 61%) and the incidence of nausea and vomiting (RR: 0.7, 95% CI: 0.46–1.06, *P* = 0.09, I^2^ = 0%).

**Conclusion:**

The administration of esketamine can reduce the incidence of emergence delirium without prolonging PACU stay time and increasing the risk of nausea and vomiting in pediatric patients. Subgroup analysis indicated that a single bolus esketamine before anesthesia induction or at the end of surgery would better reduce the risk of ED than intraoperative continuous infusion.

**Systematic Review Registration:**

https://www.crd.york.ac.uk/PROSPERO/view/CRD42024623667.

## 1 Introduction

Emergence delirium (ED) is defined as a complex of perceptual disturbances and psychomotor agitation that occurs during the early stage of post-anesthesia period (Moore and Anghelescu, 2017). The incidence of ED in pediatric patients ranges from 10% to 80%, which depends on preoperative anxiety level, age, anesthetic agents, and type of surgery (Urits et al., 2020). ED can cause adverse events such as venous catheter removal, wound bleeding, prolonged hospital stays, and even persistent emotional and cognitive changes (Shen et al., 2022). Even though ED is self-limited, strategies are essential to reduce the occurrence of ED.

Ketamine is a noncompetitive N-methyl-D-aspartate (NMDA) receptor antagonist which has been commonly used as an anesthetic in pediatric patients (Stevic et al., 2017). The effect of ketamine on ED remains a matter of debate. Previous meta-analysis has showed that, whether in adult patients or pediatric patients, ketamine is not recommended for ED prevention (Fellous et al., 2023; Ng et al., 2019). Esketamine (S-ketamine) is the S-enantiomer of ketamine with approximately two times higher potency. It also associated with faster recovery time, and fewer adverse effects than ketamine (Peltoniemi et al., 2016). Subanesthetic esketamine has been reported to have beneficial effects on postoperative pain relief and decreasing propofol requirements (Hung et al., 2024; Huang et al., 2023).

In recent years, several clinical trials have evaluated the effects of esketamine on ED in children. However, they were small-sample studies and reported controversial findings. Thus, a meta-analysis is urged to pool these data and conclude high-quality evidence. The primary outcome of this meta-analysis was to determine the effect of esketamine on the incidence of ED in children undergoing general anesthesia. The secondary outcomes were to evaluate the effect of esketamine on pediatric anesthesia emergence delirium (PAED) scale score, the Face, Legs, Activity, Cry, and Consolability (FLACC) pain scale score, post-anesthesia recovery time, and the incidence of nausea and vomiting.

## 2 Materials and methods

This meta-analysis was conducted and reported in accordance with the Preferred Reporting Items for Systematic Reviews and Meta-Analyses (PRISMA) statement and the guidelines described in the Cochrane Handbook (registry ID: CRD42024623667).

### 2.1 Search strategy

Two authors independently searched Pubmed, Cochrane Controlled Register of Trials, and Embase from inception to December 2024. To avoid the omission of relevant studies, we selected the “All Fields” option rather than “Title/Abstract.” In addition, reference lists and citing articles from included studies were screened. The search strategy was constructed using a combination of the following words: (esketamine OR S-ketamine) AND (delirium OR agitation OR restlessness) AND (child OR infant OR pediatric OR kid OR adolescent). There was no language restriction during the electronic searches.

### 2.2 Inclusion and exclusion criteria

Studies meeting the following criteria were eligible for inclusion: 1) Population: pediatric patients (aged 0–18 years) undergoing any general anesthesia procedure/surgery; 2) Intervention: administration of esketamine; 3) Comparison: placebo or blank control; 4) Outcomes: studies reporting the incidence of ED or PAED scores; 5) Study design: randomized controlled trials. Studies meeting the following criteria were excluded: 1) Case series, review papers, or retrospective studies; 2) Did not report outcomes related to ED.

### 2.3 Data extraction

EndNote X9 was used to eliminate duplicate studies. Data extraction was performed independently by two reviewers using a data extraction form. Disagreements between reviewers were resolved by discussion with a third reviewer. The following information was extracted from the eligible articles: primary author, type of surgery, patient characteristics (ages and number), dosage, route, and timing of esketamine, ED assessment methods, FLACC scores, postanesthesia care unit (PACU) stay time, and the incidence of nausea and vomiting.

### 2.4 Quality assessment

Two reviewers independently assessed the quality of the included studies according to Cochrane Collaboration’s risk of bias tool (Higgins et al., 2011), which consists of seven items: 1) random sequence generation (selection bias); 2) allocation concealment (selection bias); 3) blinding of participants and personnel (performance bias); 4) blinding of outcome assessment (detection bias); 5) incomplete outcome data (attrition bias); 6) selective reporting (reporting bias); and 7) other bias. We assigned a judgment of high, low, or unclear risk for each item. Any disagreements were resolved by discussion among all authors.

### 2.5 Statistical analysis

Data of selected studies were analyzed using Review Manager 5.3 (Cochrane Collaboration, Oxford, UK). The incidence of dichotomous data was performed using the risk ratio (RR) with 95% confidence interval (CI) and analyzed by Mantel-Haenszel method. Continuous outcomes were described as mean difference (MD) with 95% CI. Outcome data presented as a median with interquartile range were converted into mean and SD using the method recommended by Wan et al. Heterogeneity was quantified using the I^2^ statistic in all the measured outcomes. Heterogeneity was graded according to Cochrane Guidelines: I^2^ < 25% was low heterogeneity, 25%–50% was moderate heterogeneity, 50%–75% was substantial heterogeneity, and >75% was considerable heterogeneity. If I^2^ < 50% and P > 0.1, a fixed-effects model was used; otherwise, a random-effects model was selected. A *P*-value less than 0.05 was considered statistically significant.

## 3 Results

### 3.1 Study selection

**FIGURE 1:**
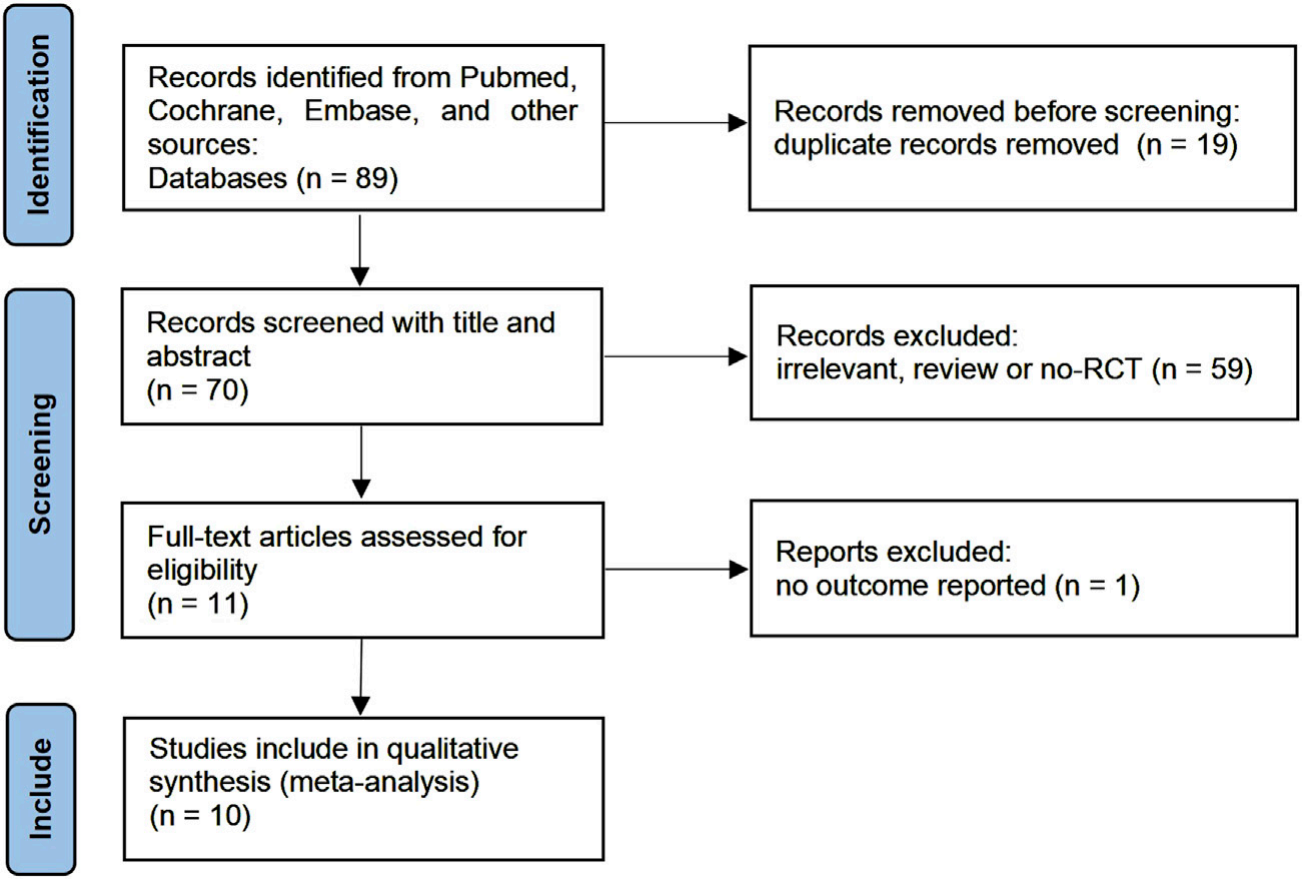
PRISMA Flow diagram.

A flow diagram summarizing the detailed steps of our study selection was described in Figure 1. Our initial search yielded 89 studies from Pubmed, Cochrane Controlled Register of Trials, and Embase. 70 studies remained after adjusting for duplicates. After screening the titles and abstracts, 59 studies were determined to be not relevant to this meta-analysis. After screening the full text, 1 studies did not report relevant outcomes. Finally, 10 studies involving 853 children were included in this meta-analysis.

### 3.2 Study characteristics

The characteristics of the included studies were presented in Table 1. All ten enrolled RCTs were conducted in China and published between 2022 and 2024. The age of the children across studies ranged from 0 to 12 years old. These studies involved tonsillectomy and/or adenoidectomy (3 studies), fiberoptic bronchoscopy (2 studies), lower abdominal or perineal surgery (2 studies), strabismus surgery (2 studies), and elective minor surgeries (1 study). Esketamine was intravenously administrated in 6 studies, of which 4 used a single bolus, 2 used a continuous infusion. 4 studies used the route of intranasal to administrate esketamine before general anesthesia.

**TABLE 1:** Characteristics of included studies.

Author (Year)	Age	Procedures	Sample size	Dosage	Route	Timing	Sedation	Measurement of ED
Chen et al. (2023)	3–7 years	Tonsillectomy and/or adenoidectomy	E: n = 54 C: n = 54	0.2 mg/kg	IV	At the end of surgery	Sevoflurane Propofol	Aono scale
Han et al. (2024)	2–6 years	Surgeries expected time less than 1 hour	E: n = 42 C: n = 42	1.0–1.35 mg/kg/h	IV	During the whole anesthesia period	Midazolam Propofol	PAED scale
Li et al. (2022)	2–7 years	Tonsillectomy	E: n = 40 C: n = 40	0.25 mg/kg	IV	At the end of surgery	Sevoflurane Propofol	RSS scale
Liang et al. (2025)	1–7 years	Lower abdominal or perineal surgery	E: n = 34 C: n = 34	0.3 mg/kg	IV	At the end of surgery	Midazolam Propofol	PAED scale 5-point agitation scale
Liao et al. (2024)	2–5 years	Tonsillectomy and/or adenoidectomy	E: n = 64 C: n = 64	0.5 mg/kg	IN	Before anesthesia induction	Sevoflurane Propofol Dexmedetomidine	PAED scale
Liu et al. (2022)	3–6 years	Strabismus surgery	E(a): n = 30 E(b): n = 30 C: n = 30	E(a): 0.5 mg/kg E(b): 1 mg/kg	IN	Before anesthesia induction	Sevoflurane Propofol	PAED scale
Lu et al. (2022)	1–6 years	Lower abdominal or perineal surgery	E: n = 29 C: n = 30	0.5 mg/kg	IN	Before anesthesia induction	Sevoflurane Dexmedetomidine	PAED scale
Xing et al. (2024)	2–6 years	Dental treatment	E: n = 36 C: n = 38	1 mg/kg	IN	Before anesthesia induction	Sevoflurane Propofol Dexmedetomidine	PAED scale
Zhang et al. (2023)	3–7 years	Fiberoptic Bronchoscopy	E(a): n = 30 E(b): n = 30 C: n = 30	E(a): 0.5 mg/kg E(b): 0.75 mg/kg	IV	Before anesthesia induction	Sevoflurane	PAED scale
Zhong et al. (2023)	≤12 years	Fiberoptic Bronchoscopy	36/36 E: n = 36 C: n = 36	0.3 mg/kg + 0.3 mg/kg/h	IV	During the whole anesthesia period	Midazolam Propofol	PAED scale

E, esketamine; C, control; IV, intravenous; IN, intranasal; PAED, pediatric anesthesia emergence delirium; RSS, Ramsay sedation scale.

### 3.3 Risk of bias

**FIGURE 2:**
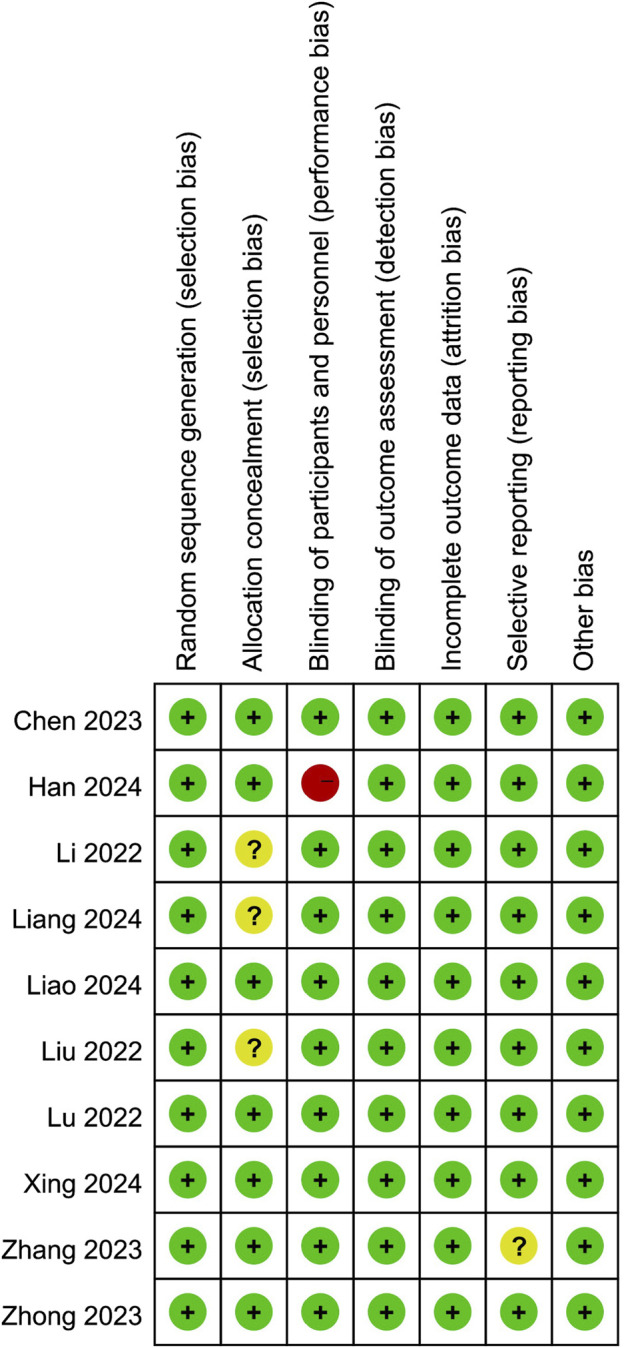
Risk of bias summary.

Risk of bias summary was outlined in Figure 2. All ten studies were randomized controlled trials and seven of them adequately reported allocation concealment. Furthermore, the participants were blinded to the intervention because most of them were preschoolers or school-aged children. Nine studies blinded the observers, and only one study described the anesthesiologist was not blinded to group allocation. Moreover, one study’s protocol was unavailable, and we considered it to have an unclear reporting bias. Last, all studies were rated low risk in attrition and other biases.

### 3.4 Outcomes

#### 3.4.1 Primary outcome

**FIGURE 3:**
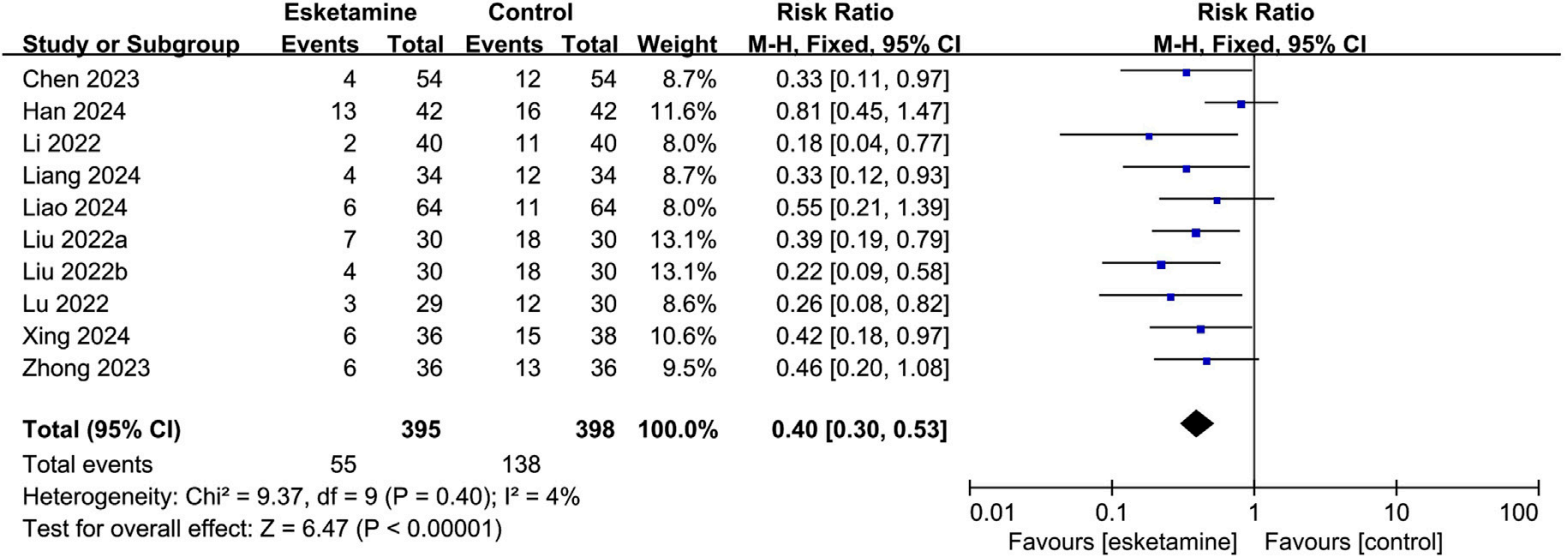
Forest plot showing the impact of esketamine on the incidence of emergence delirium.

Nine studies reported the occurrence of ED, of which seven used the PAED scale, one used the Aono scale, and one used the RSS scale. As shown in Figure 3, based on the combined data, the incidence of ED was 13.9% in the esketamine group and 34.7% in the control group. Thus, esketamine was associated with a lower incidence of ED (RR: 0.40, 95% CI: 0.30–0.53, *P* < 0.00001, I^2^ = 4%).

**FIGURE 4:**
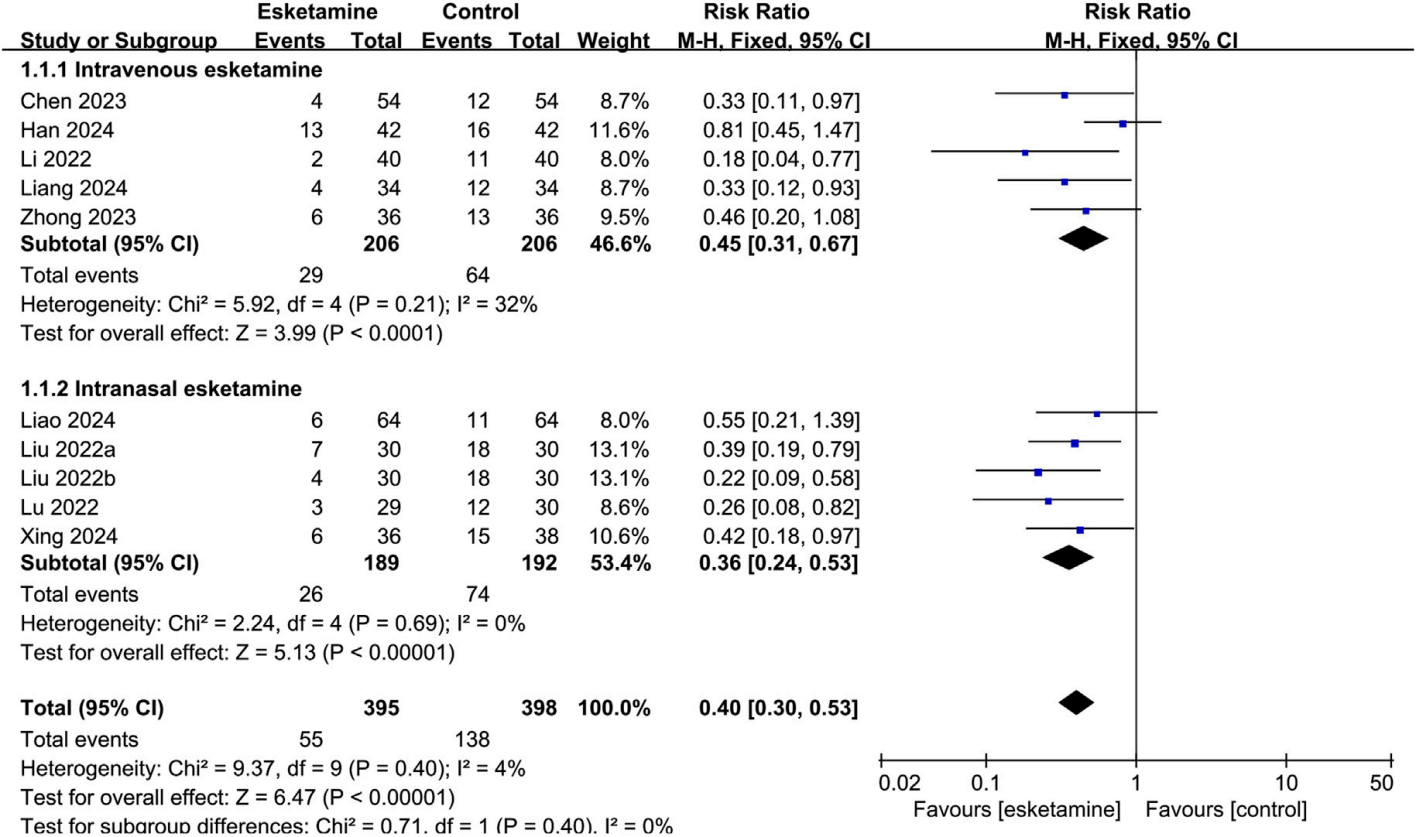
Forest plot showing the subgroup analysis of esketamine by different routes of administration on the incidence of emergence delirium.

The result of the subgroup analysis of the occurrence of ED, grouped by different routes of administration, was summarized in Figure 4. Esketamine given intravenously reduced the incidence of ED compared with the control group (RR: 0.45, 95% CI: 0.31–0.67, *P* < 0.0001, I^2^ = 32%). Intranasal administration also reduced the occurrence of ED compared with the control group (RR: 0.36, 95% CI: 0.24–0.53, *P* < 0.00001, I^2^ = 0%).

**FIGURE 5:**
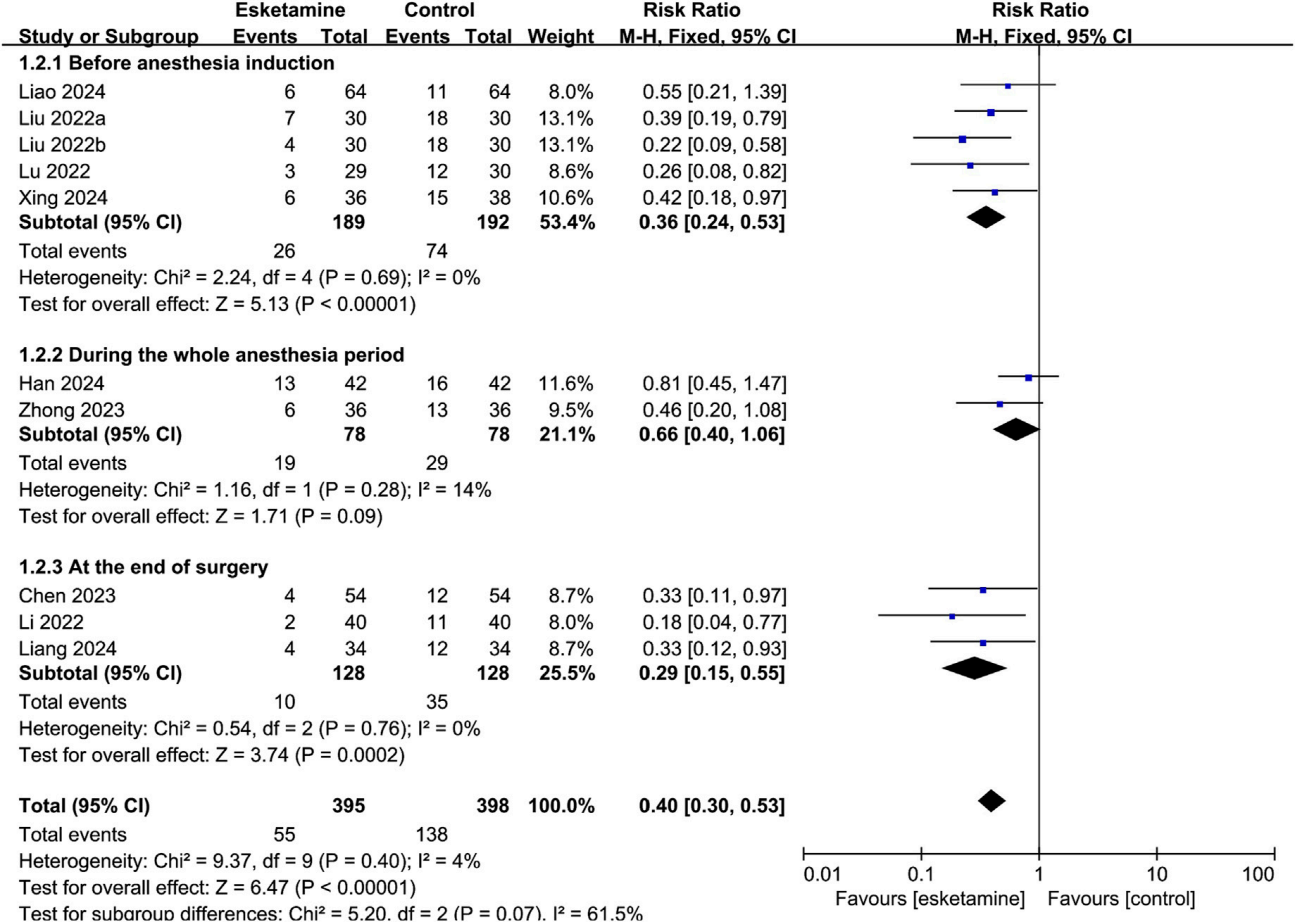
Forest plot showing the subgroup analysis of esketamine by different timing of administration on the incidence of emergence delirium.

Depending on the administration time, subgroup analysis was classified as administered before anesthesia induction, during the whole anesthesia period, and at the end of surgery. As shown in Figure 5, esketamine given before anesthesia induction reduced the incidence of ED compared with the control group (RR: 0.36, 95% CI: 0.24–0.53, *P* < 0.00001, I^2^ = 0%). Continuous infusion of esketamine during the whole anesthesia period had no significant effect on the incidence of ED (RR: 0.66, 95% CI: 0.40–1.06, *P* = 0.09, I^2^ = 14%). Esketamine given at the end of surgery reduced the incidence of ED compared with the control group (RR: 0.29, 95% CI: 0.15–0.55, *P* = 0.0002, I^2^ = 0%).

#### 3.4.2 Secondary outcomes

PAED scores were reported in nine studies. Pooled analyses suggested that esketamine reduced the PAED scores (MD: -3.66, 95% CI: -5.85–1.47, *P* = 0.001). There was considerable heterogeneity across studies (I^2^ = 99%).

Three studies reported FLACC scores. After pooling data from these studies, subjects with esketamine had lower FLACC scores than the control group (MD: -2.47, 95% CI: -3.32–1.61, *P* < 0.0001). The degree of heterogeneity was found to be considerable across these studies (I^2^ = 89%).

Six studies reported information on the length of stay in the PACU. The analysis found that children in the esketamine group had a similar stay in the PACU compared with the control group (MD: 0.5 min, 95% CI: -1.51–2.51, *P* = 0.63). Statistical heterogeneity was found to be substantial across these studies (I^2^ = 61%).

All ten studies reported nausea and vomiting, of which four observed no occurrence in both groups. Data analysis did not reveal a statistically significant difference between esketamine and the control group on the incidence of nausea and vomiting (RR: 0.7, 95% CI: 0.46–1.06, *P* = 0.09). There was no statistically heterogeneity across these studies (I^2^ = 0%).

### 3.5 Publication bias and sensitivity analysis

Funnel plot was not performed because limited number of studies reported the primary outcome (n < 10). Sensitivity analyses was applied by leave-one-out approach. The results showed consistent outcomes on the incidence of ED, which strengthened the reliability of our conclusion that perioperative administration of esketamine could reduce the incidence of ED in pediatric patients.

## 4 Discussion

The present meta-analysis with high quality of evidence showed that the application of esketamine had a significant effect in reducing the incidence of ED without prolonging PACU stay time. Moreover, the subgroup analysis indicated that a single bolus esketamine before anesthesia induction or at the end of surgery would better reduce the risk of ED than intraoperative continuous infusion.

The mechanism of ED after general anesthesia is unknown. High anxiety levels regarding surgery, separation from parents, and being surrounded by unfamiliar environment and medical staff may lead to ED (Lee and Sung, 2020). The diagnosis of ED can be based on different scale systems. The PAED scale is commonly used in pediatric patients, but the cutoff point for defining of ED remains controversial. Three of our included RCTs used a PAED score ≥10, whereas four RCTs adopted a PAED score ≥12. Previous study indicated that PAED scores over 12 appeared to provide greater sensitivity and specificity than PAED scores over 10 (Bajwa et al., 2010). Other assessment tools for ED include Richmond Agitation-Sedation Scale (RASS, 10-point), Ramsay Sedation Scale (RSS, 6-point), Riker Sedation-Agitation Scale (RSAS, 7-point), and Aono’s 4-point Scale.

Two recent meta-analyses, focused on different populations from ours, had evaluated the effect of esketamine on ED. Liu et al. (2025) included twenty-three relevant RCTs with 1996 children and reported a significantly reduced incidence of ED after esketamine administration for tonsillectomy and adenoidectomy. Another meta-analysis by Zhang et al. (2024) was performed to investigate the effects of esketamine on ED in adult patients. Their results also demonstrated that the application of esketamine during anesthesia induction significantly reduced the incidence of ED without increasing the incidence of postoperative adverse events. However, these two meta-analyses included RCTs with high risk of biases, especially on allocation concealment and blinding.

Four studies in our meta-analysis were considered to have unclear risks of selection bias or reporting bias. However, the results of these studies were consistent with our overall findings. Sensitivity analyses by leave-one-out approach further strengthened the reliability of our conclusion. Only one study by Han et al. (2024), considered a high risk of performance bias, showed that the administration of esketamine did not significantly reduce the incidence of ED. To a certain extent, the role of this bias potentially reduced the magnitude of the differences we identified.

For the severity of ED, the present meta-analysis found significantly reduced PAED scores after esketamine administration in pediatric patients. However, there existed considerable heterogeneity across the included studies. This was presumably due to the differences in types of surgery and anesthesia technique. The incidence of ED varied in different procedures. A prospective cohort study concluded that ophthalmology and otorhinolaryngology procedures were associated with an increased risk of ED (Voepel-Lewis et al., 2003). In particular, otorhinolaryngological procedures were independent risk factors for ED.

Regarding to the anesthesia technique, seven RCTs used sevoflurane for anesthesia maintenance, whereas others administrated propofol for continuous infusion. A previous meta-analysis showed that propofol-based total intravenous anesthesia (TIVA) was associated with a much lower incidence of ED than sevoflurane-based anesthesia in children (Kanaya et al., 2014). Furthermore, three RCTs in this meta-analysis premedicated intranasal dexmedetomidine in children before anesthesia induction. High quality of evidence confirmed that premedication with dexmedetomidine was associated with decreased ED (Fu et al., 2024; Sin et al., 2022).

Esketamine exhibits long-lasting analgesic effect by activating the NMDA receptor. Xu et al. (2023) found that esketamine provided a similar analgesic effect as hydromorphone in pediatric patients undergoing hypospadias. Several clinical trials had demonstrated that esketamine had anti-inflammatory property and played a protective role in postoperative cognitive function and pain management (Liu et al., 2023; Zhu et al., 2025; Han et al., 2025). In our pooled analyses, children displayed significantly lower FLACC scores when compared to the control group. Pain is a major risk factor for ED in pediatric patients (Kanaya, 2016). Adequate pain relief may contribute to the lower incidence of ED in our study.

Several limitations of the present meta-analysis should be noted. First, we only searched three major databases in our meta-analysis, which may lead to a potential omission of highly rated articles from other databases such as Scopus and Web of Science. Second, considerable heterogeneity in PAED scores made us unable to accurately conclude the relationship between esketamine and the severity of ED. Third, the ED measurement tools were inconsistent among the included studies. Even in seven studies used the PAED scale to evaluate ED, the cutoff points were different. Last, all ten studies included in the meta-analysis were conducted in China, which lacks the generalisability to promote globally. Further researches are required to validate our findings in different countries and ethnicities.

## 5 Conclusion

According to our meta-analysis, esketamine can reduce the incidence of emergence delirium without prolonging PACU stay time and increasing the risk of nausea and vomiting in pediatric patients undergoing general anesthesia. Subgroup analysis indicated that a single bolus esketamine before anesthesia induction or at the end of surgery would better reduce the risk of ED than intraoperative continuous infusion. However, high degree of heterogeneity limits the recommendations of esketamine for reducing the severity of ED and postoperative pain.

## Data Availability

The original contributions presented in the study are included in the article/supplementary material, further inquiries can be directed to the corresponding author.
